# Review on Graph Clustering and Subgraph Similarity Based Analysis of Neurological Disorders

**DOI:** 10.3390/ijms17060862

**Published:** 2016-06-01

**Authors:** Jaya Thomas, Dongmin Seo, Lee Sael

**Affiliations:** 1 Department of Computer Science, Stony Brook University, Stony Brook, NY 11794, USA; 2 Department of Computer Science, State University New York Korea, Incheon 406-840, Korea; 3 Korea Institute of Science and Technology Information, 245 Daehak-ro, Yuseong-gu, Daejeon 34141, Korea; dmseo@kisti.re.kr

**Keywords:** graph clustering, graph similarity, neurological disease, biological network, structural brain network, functional brain network, multi-layer graphs

## Abstract

How can complex relationships among molecular or clinico-pathological entities of neurological disorders be represented and analyzed? Graphs seem to be the current answer to the question no matter the type of information: molecular data, brain images or neural signals. We review a wide spectrum of graph representation and graph analysis methods and their application in the study of both the genomic level and the phenotypic level of the neurological disorder. We find numerous research works that create, process and analyze graphs formed from one or a few data types to gain an understanding of specific aspects of the neurological disorders. Furthermore, with the increasing number of data of various types becoming available for neurological disorders, we find that integrative analysis approaches that combine several types of data are being recognized as a way to gain a global understanding of the diseases. Although there are still not many integrative analyses of graphs due to the complexity in analysis, multi-layer graph analysis is a promising framework that can incorporate various data types. We describe and discuss the benefits of the multi-layer graph framework for studies of neurological disease.

## 1. Introduction

The study of neurological disorders involves a wide range of specialties and experiments resulting from various data types of various levels of detail. To understand neurological disorders more comprehensively, it is worthwhile to look at the various types of studies performed. We focus our attention on the graph analysis aspect in a wide spectrum of studies in the hopes to find a joining framework for integrative analysis that does not just involve the molecular level or the tissue level of data, but all available types of data being accumulated for the study of a neurological disorder. We focus on graph analysis methods because neurological disorders are caused by and characterized by a complex interplay of various genomic and environmental features that are often represented as graphs. Graphs are able to model the relationship between the features, as well as listing features that are important in the data analysis. Among the various graph analysis methods, graph clustering and subgraph similarity search are two of the most widely-used methods. They have been applied to study the biological data, brain images and neural signaling data in the studies of neurological disorders.

The goal of the review is to provide a wide spectrum of graph analysis applications in the study of neurological disorder. We do not attempt to summarize the finding of neurological disorders. We first look at the properties of neurological disorders and list out graph analysis measures, including graph clustering and graph similarity search, used for analyzing neurological disorders. Then, we review bio-network types and how graph clustering and similarity measures are used for causal and susceptible gene finding and disease characterization in the area of systems biology. We also review how graph analysis techniques are used to analyze structural and functional brain networks constructed from the brain images and neural signals. After a review of the existing work on graph analysis in neurological disorders, we find the need for an integrative analysis that incorporates various data sources in one analysis framework. For this purpose, we suggest that a multi-layer graph is the most appropriate data structure and further review studies on multi-layer graphs.

### 1.1. Characterizing Neurological Disorders with Graphs

Neurological disorders are characterized by an abnormality of the structure and function of the central nervous system or peripheral nervous system. There are a number of causes associated with neurological disorders, including genetic, environmental influence, physical injuries, infections and nutrition imbalance. Some neurological disorders are strictly inherited, *i.e.*, Huntington’s disease [1], while others are caused by a combination of genetic and environmental factors, *i.e.*, Alzheimer’s disease [2] and Parkinson’s disease [3]. Furthermore, neurological disorders can affect an entire neurological pathway or a single neuron, and such dysfunction can be quite diverse. Thus, various perspectives of neurological diseases are studied, and various bio-medical data types, including the genomic data, bio-specimens and brain images, are generated. These data usually contain features that have a complex relationship, and graphs are often used to capture these complex relationships.

In systems biology, relationships between biological components are represented as a graph, such as Protein-Protein Interaction (PPI), disease-gene association, metabolic pathways, biochemical networks and regulatory networks. The biological network (bio-network) studies the biological system at the genetic and molecular level. This is essential for understanding the causes and mechanisms of disease progression and, thus, aiding in better treatments and novel drug developments. On the other hand, graphs are also frequently used to model and analyze the anatomy and function of brain [4,5]. Brain networks generated from a variety of diagnostic brain imaging and signaling techniques are used for neurological disease. These brain analyses help to monitor the resulting changes in brain structure and function and how they ultimately shape behavior.

### 1.2. Graph Clustering and Graph Similarity

A graph structure is a representation of a set of entities; these entities constitute the nodes that represent the biological components and edges that describe the association between pairs of nodes. Biological and brain network analysis often involves graph clustering and graph similarity. Analysis of bio-networks using graph clustering and subgraph identification is often applied to understand the complex pathology and to address the important translational challenges. This is also applied in brain networks to improve the understanding of the complex brain structure and of the complex functional relationship between sub-regions of the brain.

Graph clustering methods and module detection methods group the nodes into clusters, or modules, on the basis of graph topology, such that the resulting clusters have high intra-homogeneity/connectivity and low inter-homogeneity/connectivity among the sub-graphs formed. Four commonly-used graph clustering algorithms are the Markov Cluster Algorithm (MCL) [6], Molecular COmplex DEtection(MCODE) [7], Highly Connected Subgraphs (HCS) [8] and Restricted Neighborhood Search Clustering (RNSC) [9]. MCL is based on flow simulations in graphs and works to increase the contrast between regions of high and low flow, by evaluating the successive power of the adjacency matrix. The algorithm converges, resulting in a graph partition with high flow regions separated from regions with no flow. The MCL algorithm is widely applied on protein-protein interaction networks [10,11]. The MCODE algorithm is designed to detect densely-connected regions in PPI for the purpose of predicting protein complexes [12]. Initially, MCODE assigns weights to the vertices based on their density, *i.e.*, local connectivity. It then selects seed vertices based on the weights as the initial clusters. These seed vertices are further augmented by outward traversal of the isolated dense regions in accordance with preset parameters. The HCS algorithm recursively finds the minimum graph cut that leads to a graph partition that outputs highly connected components or subgraphs. HCS have been applied for gene expression analysis [13]. RNSC is a partition-based algorithm that starts with a random cluster assignment and proceeds by reassigning nodes to clusters. The quality of the clusters thus formed is then evaluated using a cost function. The final obtained clusters are filtered based on their size, density and functional homogeneity.

Subgraph isomorphism, given a pair of graphs *A* and *B*, is defined as a problem of determining whether graph *A* contains a subgraph that is isomorphic, *i.e.*, identical in structure, to *B*. Subgraph similarity is a relaxation of this problem that instead of determining a match or no match, determines the similarity of the subgraph measured by a “similarity score”. The core of the graph similarity search is the similarity scores that each method proposes. Three commonly-used subgraph theoretical algorithms are GraphGrep [14], NetworkBlast [15] and SAGA [16]. GraphGrep is a hash indexing-based method for subgraph matching, which allows efficient filtering by selecting the most relevant subgraphs from the relevant graphs. NetworkBlast has a graph similarity model that is designed for comparing and analyzing multiple protein networks. The log likelihood ratio scoring method was used to evaluate the subnetwork fit to the desired structure. SAGA is an approximate subgraph matching technique based on node gaps, node mismatch and graph structural differences. The distance measure is used for matching the subgraph, where the measures include StructDist to measure the structural difference, NodeMismatches that estimate the penalty for the mismatch of labels and NodeGaps that compute the penalty on the gap nodes.

Graph theoretical measures can be used as similarity values in the subgraph similarity problem, or they can be used to characterize graphs. The two most common graph theoretical measures are the clustering coefficient and characteristic path length, which are often used to distinguish between regular, random and small-world networks [17]. The small-world network refers to a network for which the mean shortest-path distance between nodes increases slowly as a function of the number of nodes in the network. The small-world structure is hypothesized to reflect an optimal situation associated with rapid synchronization and information transfer [18], minimal wiring costs, as well as a balance between local processing and global integration [4]. The clustering coefficient is defined as the ratio of the number of existing connections among the node’s immediate neighbors to all of their possible connections. It defines the local efficiency of the information transfer of a network. The characteristic path length quantifies the average minimum number of connections that link any two nodes. It defines the global efficiency, indicating the capability of the parallel information propagation of a network. There are also centrality measures that identify important nodes in a network, such as hubs [4]. Depending on the characteristic of “important” nodes, different centrality measures can be used. The degree centrality of a node computes the number of edges extending from it. The closeness centrality of a node computes how close it is to all of the other nodes. It is defined as the inverse of the sum of distances based on the length of the average shortest path between a node and all nodes in the graph. The betweenness centrality of a node on the other hand measures how many time the node was part of a communication path between a pair of nodes. Other local measures, including eccentricity, *i.e.*, the maximum distance between that node and any other node of the graph, and radiality, *i.e.*, node centrality index, can also be used to find significant nodes. There are also other global measures, including modularity and the minimum spanning tree of a graph. Modularity is a global measure of the network that measures the separability of nodes to modules. A network with high modularity is able to group nodes to modules with high intra-module node connectivity and low inter-module node connectivity. The minimum spanning tree of a network is also being extensively used to characterize the topology in more recent studies. It has been shown to avoid several methodological biases in the study of brain networks [19]. Table 1 summarizes the graph theoretical measures and the formulae for computing them.

## 2. Types of Bio-Networks and Applied Analysis on Neurological Disorders

In this section, we first look at various types of bio-networks and publicly available resources. Then, we review how these bio-networks are analyzed to study the neurological disorders in two sub-categories of problems: causal and susceptible gene finding and disease characterization.

### 2.1. Types of Bio-Networks

There are several types of bio-networks. We classify them as gene networks, protein-protein interaction networks and biological pathways. We describe each type and provide a brief summary of publicly available databases for the biological data in Table 2.

Gene networks model the various types of associations among the genes. A gene network is an undirected graph with nodes representing genes and edges representing a type of association. A gene co-expression network is a kind of gene network in which the edges denote the correlation in the expression patterns of the genes [21]. Gene networks constructed from the analysis of expression patterns are often associated with specific conditions of the conducted studies. Another variant of a gene network is the gene-disease network, which has been extensively studied in the past few years. In a gene-disease network, nodes can also represent the various disease types, and edges denote the associations of gene to diseases. Gene networks are used to study complex neurological disorders, including autism [22] and Alzheimer’s disease [23].

The physical interaction among proteins constitutes a large part of the physiology of living things. PPI networks represent proteins as nodes, and the physical interactions or functional associations between the proteins are represented as an undirected edge. Proteins play a central role in biological function, and their interactions control the mechanism, which may lead to healthy or disease states. Many disease states result due to the change in protein functioning, *i.e.*, change in PPI topology. Experiments, such as yeast-two-hybrid screening, affinity purification coupled to mass spectrometry, co-immunoprecipitation and chemical cross-linking, have been used to determine the PPI. Furthermore, the indirect approach of text mining methods that mine the co-occurrence of gene names in the literature have also been used to construct PPI. A detailed description of PPI can be found in a review by Rivas *et al.* [24].

Biological pathways are small to medium-sized directed bio-networks representing the chain of actions among bio-molecules that lead to a certain functionality, such as the production of other biomaterial or that trigger changes in cellular content. Most pathways are manually curated results of accumulated information from several experiments. Thus, although smaller in size, they have a more complex form. The nodes in the graph denote genes, proteins, metabolites, small molecules, reactions, chemical compound, diseases or drugs. Edges in this bio-network show various biological reactions, such as a modification in gene expression, alteration in a protein or other biochemical reactions. There are various types of biological pathways, including metabolic pathways, regulatory pathways, signaling pathway and disease pathways. Metabolic pathways represent the series of chemical reactions in a cell. A node of the metabolic pathways represents an enzyme (protein) that catalyzes the reactions, a metabolite (initial chemical compound), a product of a substrate (intermediate chemical compound) of enzymes or other co-factors. Regulatory pathways describe the regulation of gene activities. Nodes of regulatory pathways are often composed of transcription factors (proteins) and DNA (genes). Regulatory networks are a general form of regulatory pathways that can be either directed or undirected and are often composed of transcription factors and genes to which they bind. High-throughput technologies for obtaining the binding information include ChIP-chip and ChIP-seq. Signaling pathways describe the complex signal transduction process that integrates information from PPI, regulatory pathways and metabolite pathways. Disease pathways represent the relationships among the critical components, including genes, regulators and metabolites, of a disease. The Alzheimer’s disease pathway outlines the mechanisms of the formation of amyloid plaques and neurofibrillary tangles [25]. The Kyoto Encyclopedia of Genes and Genomes (KEGG) pathway displays current genes, proteolytic events and other processes associated with the progression of Alzheimer’s disease [26].

### 2.2. Bio-Network-Based Neurological Disorder Analysis

The applications of bio-network analysis for neurological disorders can be largely categorized into finding causative and susceptible genes and disease characterization. We selectively review studies to show the methodological diversity in each category and summarize their characteristics from the perspective of the analysis approaches.

#### 2.2.1. Causal and Susceptible Gene Finding

Graph clustering methods and local graph theoretical measure for finding significant nodes have been dominantly used in the studies of causal and susceptible gene finding in neurological disorders.

The abundance of molecular interaction network data turns out to be particularly powerful for disease gene prediction [42]. These bio-networks provide the basic framework of the cellular processes, and graph analysis helps to model the complex interactions among multiple genes and their higher level organizations. The closely-connected genes are assumed to have similar functions in bio-networks. With this assumption, the functional annotation of genes or unknown proteins can be predicted. Lopez *et al.* [43] observed that Alzheimer’s disease-related genes are highly interconnected. Based on this observation, they used a graph clustering approach to find novel Alzheimer’s causative and susceptible genes. To do this, a combined bio-network was initially constructed by considering 22,194 interactions between 8347 proteins derived from databases, such as DIP, IntAct, MINTand HPRD. The Markov clustering algorithm [6] was then used to identify the cluster representing functional modules. Similarly, Diao *et al.* [44] performed a comprehensive gene-level assessment to search important molecular markers and pathways of Parkinson’s disease. The correlation network for Parkinson’s disease was identified by applying the DPClus [45] graph clustering algorithm. The densely-connected nodes of the cluster were assessed by associated GO terms and KEGG pathways. The approach resulted in finding significant pathways, such as the Parkinson’s disease pathway.

Talwar *et al.* [46] also applied graph clustering to find novel genes in Alzheimer’s disease. However, instead combining existing networks, they generated a consensus PPI from a PPI network generated from three different data type, *i.e.*, genome-wide linkage analysis, genome-wide association studies and genome-wide expression profiling, to identify the candidate genes involved in Alzheimer’s disease development and progression. The consensus PPI was constructed by integrating Alzheimer’s disease linkage, genetic association and gene expression data proceeding by modeling PPI by identifying overlapping genes in the PPI generated form the three sources ranked by cumulative rank score. The MCL clustering of the consensus PPI comprised of 640 nodes and 2214 edges resulted in six significant clusters with seven genes forming the central hub nodes. The majority of top ranked candidate genes found were shown to be associated with the molecular mechanisms and pathways of Alzheimer’s, which may be crucial for predicting Alzheimer’s risk. Graph clustering in the bio-network is also used to determine the molecular markers and significant pathways.

Local characteristics of networks are also used for finding significant genes. Guney *et al.* [47] proposed a gene prioritization approach for a multiple gene-phenotype association and interaction dataset. For Alzheimer’s, a disease-association score was assigned to the genes in the PPI. The score was computed by determining multiple shorter paths between nodes. The path with more gene association was considered shorter as compared to others. The score helped to evaluate the biological significance of the neighbors. The network was constructed from 11,250 proteins, and the top 1% of the protein, *i.e.*, 116 proteins uniquely mapped to gene for Alzheimer’s, was identified.

Winkler *et al.* [48] considers both graph characteristic, as well as graph clustering methods to find the roll of sex steroids in the degeneration of hippocampal neurons in Alzheimer’s disease. They considered graph theoretical measures as closeness centrality, eccentricity and radiality to determine the crucial, highly-centered nodes in the PPI network of Alzheimer’s to find causal genes. These measures were used to compute the shortest paths between nodes in the graph. The resulting two highly-connected nodes identified were the Androgen Receptor (AR) and the estrogen receptor alpha (ESR1). Furthermore, applying MCODE [7], a graph theoretic clustering algorithm, they identified five dense subgraph. Three subgraphs were composed of transcription factors that belong to the same protein subfamily, and two subgraphs contained kinases and ligases in the signal and degradation pathways.

Whether obtaining data by publicly available bio-networks, by generating a new bio-network or by combining existing networks with new data, the methods used for gene finding are dominated by graph clustering methods and significant node finding through local graph theoretical measures.

#### 2.2.2. Disease Characterization

There are several approaches for characterizing a disease. Predicting new functions of genes or proteins in the disease pathway, finding abnormal interactions in the bio-network of patients compared to healthy individuals and the analysis of the graph properties of the bio-network of patients are a few ways that have been successful.

**Figure 1:**
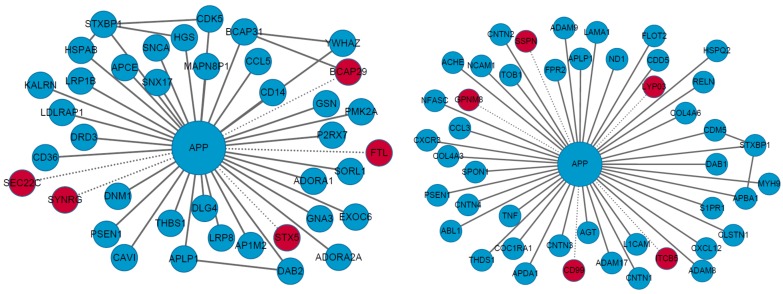
APP protein-protein interaction sub-networks. Red nodes represent the proteins from Yeast Two-Hybrid screening and blue nodes indicate interactors extracted from the databases. Adapted from “Amyloid precursor protein interaction network in human testis: sentinel proteins for male reproduction”, 2015, BMC Bioinformatics, 16:12, p. 5. Copyright 2015 Silva *et al.* [49]; licensee BioMed Central.

Bio-networks are analyzed to find new functions of proteins involved in neurological disorders in the work of Silva *et al.* [49]. They analyzed Amyloid Precursor Protein (APP) to determine its involvement in several biological functions, such as male fertility, cell adhesion, cell motility, signaling and apoptosis. The clustering coefficient was used to characterize the APP network, and betweenness centrality and closeness centrality were used to determine the relevant proteins involved in the pathways. Figure 1 shows the APP interactors involved in cell adhesion extracted from the extended APP/APLP2 network. It shows the APP interactors involved in vesicle-mediated transport extracted from the extended network. Different colors are used to denote interactors extracted from different sources; red nodes denote proteins from the yeast-two-hybrid screen; whereas blue nodes from the databases. The work provides an insight about the interactions of key APP proteins and on the function of APP in the male reproductive system.

Functional summaries of disease-specific bio-networks can also characterize the disease of interest. Seah *et al.* [50] proposed a Functional Summary Generator (FUSE), which generates functional maps using graph theoretical analysis that enables the investigation of the higher level organization and modularity within the PPI. FUSE is a greedy approach based on the profit maximization problem [51]. It first determines the functional clusters and then iteratively selects clusters that result in an increased profit. They considered Alzheimer’s as a case study and constructed low- and high-resolution functional summaries that help to understand process-process interactions. The low-resolution summary gave a functional overview of the processes related to the disease, whereas a high-resolution summary provides the in-depth functional landscape of the disease, revealing associations between processes related to the disease.

Unusual PPIs have been implicated in a number of neurological disorders, such as Parkinson’s disease [52], Alzheimer’s disease [53], autism [54] and Huntington’s disease [55]. Hence, detecting abnormal PPI can give a better insight into the root cause of these diseases. For example, Li *et al.* [56] proposed a systems framework involving the interactome, gene expression and genome sequencing to identify a protein interaction module with members strongly enriched for autism candidate genes. They constructed the PPI network using the human protein interactome from BioGrid comprising 13,039 proteins and 69,113 curated interactions. The network was clustered by Blondel *et al.* [57], which resulted in smaller modules. To determine the associations of the network modules with autism spectrum disorder, they first considered the curated genes implicated in Autism Spectrum Disorder (ASD); among a total of 484 genes in the database (https://gene.sfari.org/autdb/), 383 were on the protein interaction network. Enrichment tests for each module in the network revealed that for some specific modules’ *de novo* copy number variations, the rare copy number variations and the disruptive mutations each displayed a significant enrichment.

**Figure 2:**
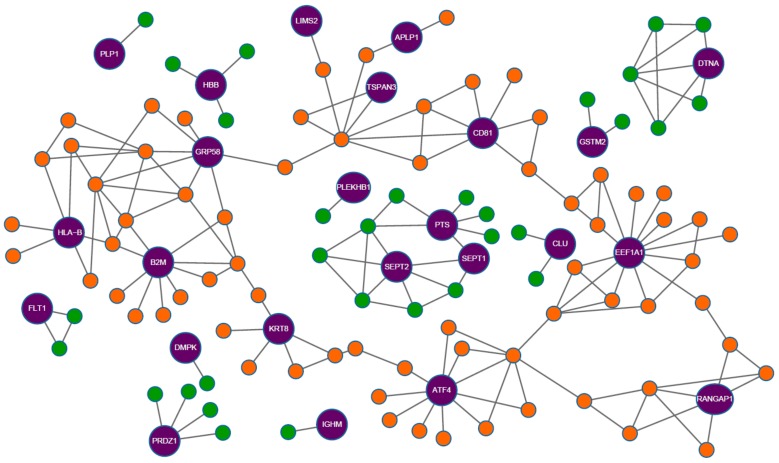
The Alzheimer’s brain network showing connectivity of seed proteins. Purple nodes indicate the seed-proteins with their name. Orange nodes indicate neighboring proteins that belong to the giant component, *i.e.*, the largest section of a network whose nodes are connected. Green nodes indicate neighbors that are not included in the giant component. Adapted from “A computational analysis of protein-protein interaction networks in neurodegenerative diseases”, 2008, BMC Systems Biology, 2:52, p. 7. Copyright 2008 Goni *et al.* [58]; licensee BioMed Central Ltd., London, U.K.

Graph characterization of a disease-specific bio-networks is also important for understanding diseases. Go*ñ*i *et al.* [58] aided the understanding of the basis of Alzheimer’s Disease (AD) and Multiple Sclerosis (MS) by considered PPI and gene expression to study the centrality-related features of proteins whose genes were differentially expressed (seed proteins) with respect to their protein neighbors. Four disease-specific bio-graph networks were used: an MS network from blood tissue (MS-blood), an MS network from brain tissue (MS-brain), an Alzheimer’s network from blood tissue (AD-blood) and an Alzheimer’s network from brain tissue (AD-brain). The main topological properties considered were the average degree and the betweenness centrality, which were analyzed using the shortest path’s degree and the cluster coefficient. Figure 2 shows the Alzheimer’s network constructed from the brain tissue with 25 seed proteins and 109 neighbors. The purple-colored nodes indicate the seed protein; nodes in orange denote neighbor proteins whose nodes are connected either directly or indirectly; and nodes in green represent neighbor proteins that are not part of the considered network. They observed that there were only two direct interaction links between the seed proteins among the total 191 links. The results presented indicated that both diseases shared common characteristics as the lowest average degree of seed-proteins and a higher degree of betweenness. Their finding also shows that the seed proteins in peripheral regions of the PPI map are involved in different pathways and are integrated into subnetworks of the complete human proteome network.

## 3. Types of Brain Networks Used in the Studies of Neurological Disorder

Brain images and neural signals are another major type of data used to study neurological diseases. Brain images or signals are often segmented into subsections, and their associations are often represented as brain networks (or biological neural networks). In this section, we first briefly review how such brain networks can be constructed from the brain image or signal data in general and review specific network construction processes and analysis methods applied in each study of neurological disorders.

### 3.1. Types of Brain Networks

There are two major types of brain networks: structural brain networks and functional brain networks. In structural brain networks, edges connect modules that are structurally connected; while in functional brain networks, edges connect modules that are functionally associated. There are no central depositories for brain networks for neurological disorders, since brain network construction is dependent on the types of experiments performed. In the following, we look at general network construction processes and experiments that generate the data for each type of network.

#### 3.1.1. Functional Brain Networks

A functional brain network is derived from physiological observation of brain activities through brain signals or brain images. The edges of the functional brain network represent the functional connectivity between nodes that represent either anatomical segments or voxels in brain images [59]. Functional connectivities are determined by statistical dependencies among remote components measured in various neuro-physiological events. Furthermore, functional connectivity is dynamic in nature, that is it is highly time-dependent and changes frequently in milliseconds being modulated by sensory stimuli and task context.

There are two types of techniques to derive dynamic functional events: electro-magnetic techniques and hemodynamic techniques. Electro-magnetic techniques measure post-synaptic current flow created by magnetic fields that can be recorded outside and inside the skull that have high temporal resolution, but low spatial resolution. Hemodynamic techniques measure the blood follow in the brain, which results in high spatial resolution, but poor temporal resolution. Electroencephalography (EEG) and Magnetoencephalography (MEG) are two representative non-invasive electro-magnetic techniques. EEG measures the change in electrical signals as the clusters of neurons become active, and MEG measures the change in magnetic fields relative to the change in electrical activity. intracranial Electroencephalography (iEEG), also called Electrocorticography (ECoG), is an invasive electro-magnetic technique that monitors the electrical activity of the cerebral cortex, which uses electrodes placed directly on the exposed surface of the brain. Compared to EEG, iEEG and MEG have better spatial resolution. iEEG has the obvious disadvantage of being invasive. functional Magnetic Resonance Imaging (fMRI) and Positron Emission Tomography (PET) are the two representative hemodynamic techniques. fMRI measures the brain activity during rest or while doing tasks by the correlated fluctuations in the Blood Oxygen Level-Dependent (BOLD)-signal with respect to time. Blood is more oxygenated in an active region of the brain, and the difference in the magnetic susceptibility between oxyhemoglobin and deoxyhemoglobin is detected by MR. PET is a test that uses a radioactive chemical tracer that is traced by a special camera that tracks the positrons, *i.e.*, positively-charged particles, that are emitted by the tracer. Although PET has high spatial resolution, because of the use of radioactive materials, it is hard to find human subjects.

**Figure 3:**
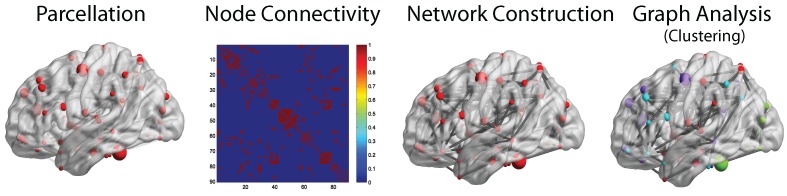
Overview of brain network analysis. Clusters are color coded in the rightmost figure.

Among the listed experiments, the most popular experimental measure for the functional study of brains is through fMRI. Crosson *et al.* [60] presents the specific advantages of fMRI over other functional imaging techniques. Three advantages of fMRI are as follows: fMRI is non-invasive and does not expose the subject to radiation; fMRI shows an image activity in deep, subcortical structures, whereas this is difficult if not impossible with EEG- and MEG-based techniques; and in fMRI, the same platform used to acquire functional images can be used to acquire high resolution anatomic images. The general fMRI studies often measure the brain signals given a certain task, but recent studies also apply the fMRI in the resting state to find patterns in neurological disorders. Brain imaging studies have suggested that the brain is in a state of activation even during the resting period. These resting state fMRI (rs-fMRI) data have been applied to a number of different problems in neuroscience, which include diseases, such as Alzheimer’s [61,62], Parkinson’s [63] and schizophrenia [64]. A detailed review of rs-fMRI is presented by Heuvel *et al.* [65]. Figure 3, shows a general overview of functional brain network analysis, where the brain image data are first subjected to parcellation that divides the brain into a number of regions or parcels with homogeneous characteristics. The functional connectivity matrix forms a full symmetric matrix between elements (voxels, neurons and recording sites) that provides a simple characterization of functional interactions. The network constructed by the functional connectivity is analyzed by the graph clustering approach to find similarity clusters. A detailed review of the construction of functional brain networks can be found in [66,67,68,69,70].

#### 3.1.2. Structural Brain Networks

A structural brain network is derived from anatomical observations from diffusion-based neuro-imaging data, such as diffusion tensor imaging or diffusion spectrum imaging [71]. The edges denote anatomical connections between sets of neural components. More specifically, they refer to the connectivity of axons. Determining the existence of the connectivity is based on the delineation and subsequent assessment of white matter fiber tracts within the brain

The network of structural connectivity in the human brain can be constructed by using structural Magnetic Resonance Imaging (MRI) and diffusion MRI. MRI is a widely-used imaging technique that uses strong static magnetic fields to line up and fall back the nuclei of hydrogen atoms, sending out radio waves that are detectable. Diffusion MRI, also referred to as Diffusion Tensor magnetic resonance Imaging (DTI), is a more recent method that measures the diffusion of water molecules in white matter fibers in the brain, called anisotropic diffusion. In DTI, unlike MRI, which often focuses on the grey matter, white matter fiber tractography is used to build the structural brain network. An assumption states that more directionally-dependent water flow indicates the presence of more axons running through the underlying white matter and, thereby, a greater structural connectivity. The network formed can be analyzed for the structural characteristics. The most common measure used along DTI is Fractional Anisotropy (FA), which specifies the directional dependency of water diffusion in the brain. These measures help in understanding the abnormalities that may be present in the brain network [72]. A detailed review of the construction of structural brain networks can be found in [66,73,74].

### 3.2. Graph Analysis Applications on Brain Networks

Studies of neurological disorders based on brain images and signals are closely related to the types of experiments performed and, thus, to the types of brain networks constructed and analyzed. The most prevalent types of brain network studies are functional brain networks followed by structural brain networks. In the following, we review studies of neurological disorders classified by the types of brain networks that are analyzed.

#### 3.2.1. Analysis of Functional Brain Networks

In most of the functional brain network analyses, the topological characteristics of the networks of patients in comparison with the healthy individual are analyzed through graph theoretical measures. We look at functional brain graph analysis applied to Alzheimer’s disease, Parkinson’s Disease (PD), MS, Autism Spectrum Disorder (ASD), epilepsy and Attention Deficit Hyperactivity Disorder (ADHD).

The difference in the global topological features of functional brain networks for Alzheimer’s patients compared to the control was analyzed by Supekar *et al.* [62] and Stam *et al.* [4]. In the work of Supekar *et al.* [62], functional graphs were constructed based on the correlation matrix for the anatomical region extracted from wavelet analysis of the rs-fMRI data. In their graph, the edges between the nodes are determined based on a threshold value for wavelet correlation, *i.e.*, edges are formed between two nodes with a wavelet correlation higher than the threshold. The clustering coefficient and characteristic path length were used to characterize the constructed functional graph for Alzheimer’s patients and the healthy control. They observed that the Alzheimer’s patients showed lower clustering coefficients, but similar characteristic path lengths compared to controls, which suggests disrupted global functional organization. They suggested that the graph analysis measures might be useful as an imaging-based biomarker to distinguish Alzheimer’s disease from healthy aging. Similarly, Stam *et al.* [4] analyzed the changes in the large-scale graph structures of resting-state brain. They used MEG-based images of both cases and controls to generate a functional brain network and then computed graph topological measurements, such as the clustering coefficient and path length, to characterize how the brain functions differently from case to control. The graph consists of 149 nodes, where each node represents the matching MEG channel, edges represent the link between the pair of channels and the edge weight denoted the phase log index values between all pairs of MEG channels. The results show that patients with Alzheimer’s disease had reduced connectivity shown by decreased clustering, similar to Supekar *et al.* [62], in addition to increased path length. The general pattern that emerged from their study showed that the bio-networks associated with various types of brain disease, such as Alzheimer’s disease, schizophrenia, brain tumors and epilepsy, are closer to random networks, and healthy networks are closer to small-world networks.

Multiple Sclerosis (MS) is another neurological disorder that is studied by brain networks. Tewarie *et al.* [75] analyzed the functional brain network of MS patients compared to healthy controls using MEG images. To construct the function network, initially, the sensor channel data of MEG were projected onto the Automatic Anatomical Labeling (AAL) atlas using beamforming that resulted in 78 nodes with time series data mapped. Beamforming or spatial filtering is a signal processing technique used in sensor arrays for directional signal transmission or reception. Next, the adjacency matrix was constructed for each frequency band separately based on the functional connectivity between each pair of nodes or time series. The Phase Lag Index (PLI) was used [4], which calculates the asymmetry of the distribution of (instantaneous) phase differences between the two time series. Subsequently, Kruskal’s algorithm [20] was applied to obtain Minimum Spanning Trees (MSTs) to measure the functional connectivity. A comparative analysis was carried out between healthy controls and MS patients by finding the dissimilarity between the respective MSTs using the information theoretic dissimilarity measure that calculates the information change between the two MSTs. The analysis showed a decrease in global integration and hierarchy, which explains the reduced cognitive performance in MS. The finding indicates that MST analysis helped to detect network changes in the core of functional brain networks for MS patients. Moreover, they were able to identify functional brain network differences between early MS patients and healthy controls.

Parkinson’s disease is another widely-studied neurological disorder. G*ö*ttlich *et al.* [63] and Sang *et al.* [76] investigated the difference of Parkinson patients against the controls in the rs-fMRI-based functional networks. Networks generated by G*ö*ttlich *et al.* [63] and Sang *et al.* [76] were constructed in a similar manner. The nodes were obtained by segmentation of the brain functional network using the Automatic Anatomical Labeling (AAL) atlas [77]. Edges were obtained by measuring the temporal correlation between each paired node, calculated by the Pearson correlation coefficient in the time series acquired for each node. A threshold was selected to mark an edge between the nodes. If the absolute correlation coefficient was higher than the threshold, an edge is formed. G*ö*ttlich *et al.* [63] measured the cluster coefficient on a local level and the characteristic path length at the global level to characterize the obtained functional brain networks. They observed that the increased characteristic path length indicates a less efficient organization of the brain network for Parkinson’s patients. The finding indicates reduced efficiency in the brain network topology of patients as compared to controls. Sang *et al.* [76] focused on the topological characteristics of the large-scale functional brain network in early-stage Parkinson’s patients. Their analysis showed that early-stage Parkinson’s patients had a significant decrease in global efficiency, but no significant difference in the local efficiency of the brain network as compared to the control. Significantly decreased global efficiency was associated with decreased long-range connections across remote cortical regions in Parkinson’s patients. This decreased global efficiency indicated a reduced capacity of information transfer across the entire brain. Dubbelink *et al.* [17,78] used MEG data to characterize the functional brain network of Parkinson’s in different stages or clinical measures of disease progression. In their studies, clustering coefficients, averaged shortest path lengths and MST were to characterize the constructed networks. They showed that impaired local efficiency, *i.e.*, the inverse of the average shortest path connecting all neighbors of a node, and network decentralization are early features of Parkinson’s disease. As the disease progresses with time, changes in brain functional graph topology accumulate and result in reductions in global efficiency, *i.e.*, the inverse of the average shortest path, which have a close association with the deteriorating cognitive and motor function. The results of Dubbelink *et al.* [17] show that the early-stage non-medicated patients were characterized by lower clustering coefficients, but preserved path lengths, which indicates reduced local integration with a preserved global efficiency of the brain network in the early motor stage of the disease. The results seemed similar to the one obtained from Tewarie *et al.* [75] and Dubbelink *et al.* [17] for MS indicating that brain networks move towards a more random network organization in both disease.

Other neurological diseases, including tuberous sclerosis complex, epilepsy and attention deficit hyperactivity disorder, have also been studied by constructing functional brain networks. Peters *et al.* [79] analyzed functional connectivity through EEG coherence in a large sample set of children with Tuberous Sclerosis Complex (TSC), a disorder with a high prevalence of autism spectrum disorder. An undirected weighted graph was built using the 19 electrodes as nodes and inter-electrode coherence values as edge weights. The graph analysis for autism spectrum disorder showed the absence of a higher clustering coefficient and of a longer path length despite the decrease in long-over-short range coherence. The result shows that the nodes are spatially more clustered, but are not functionally clustered, indicating an altered network topology. These altered network topologies in TSC represent a functional correlate of structural abnormalities and may play a role in the pathogenesis of neurological deficits. Ortega *et al.* [80] analyzed iEEG data for the localization of the Epileptogenic Zone (EZ), also called focus, responsible for seizures or the ictal state in epilepsy patients. Recurrent seizures are characteristic of epilepsy, and the localization of the seizure sites is crucial for the treatment and prevention of the spread of ictal activity in different regions of the brain. The localization of the focus is done by identifying the crucial nodes in the iEEG-based functional brain network. In the functional network, each node represents electrodes’ time series data, and the edges are weighted by the Pearson correlation coefficient between the two electrodes. The crucial nodes used for localization of the focus are selected to be the nodes with the highest local synchronization power, the most connectedness and the highest seizure interaction load. This approach helped to identify nodes that seem relevant from the global interaction perspective, one being the most connected node, *i.e.*, the node with the highest number of links, and other with the highest load, which is computed by the node betweenness centrality measure. Using these observations, they address the question of whether removal of these nodes during surgery is crucial in the suppression or reduction of the quantity of postoperative seizures. The findings for five ECoG records show that local areas with high synchronization power appear to be significantly involved in the development of epileptic seizures. Hu *et al.* [81] evaluated the structural symmetry of the weighted brain network for Attention Deficit Hyperactivity Disorder (ADHD) using graph isomorphism. The brain network was built using resting state fMRI (rs-fMRI) data for ADHD. The graph was constructed by first preprocessing of rs-FMRI data and then extracting the anatomical regions of interest using the AAL atlas. The nodes denote the anatomical region, and the edges represent the functional connectivity between the nodes. Isomorphism was used to investigate the structural symmetry of every node pair in a weighted brain network. For a given weighted graph, the symmetry between the two nodes was defined based on the isomorphism level of the residual graphs of those two nodes, and the isomorphism approximation error was computed using the suboptimal eigen decomposition algorithm by Umeyama [82]. The experimental results indicated that for the inattentive type of ADHD subjects, higher network symmetry was observed as compared to the typically development of children.

Many of the functional brain network analysis of existing studies on neurological disorders observe an altered network topology compared to healthy individuals. In the case of Alzheimer’s, similar to the abnormal topological observation in the structural brain network, the functional brain network analysis also shows disrupted organization. The graph theoretical measure on the functional brain network reveals that the small-world metrics can characterize the functional organization of the brain in Alzheimer’s disease. Most of the studies on Alzheimer’s showed that an increased path length has been interpreted to result due to the loss of connectivity. The modularity graph metric for Alzheimer’s indicated a decrease in value for the beta (13–30 Hz) and gamma (>30 Hz) band, implying decreased connectivity due to loss of connector hubs. The functional brain network analysis for MS using the minimum spanning tree measure shows that the altered functional networks in the theta and alpha2 frequency bands of time series data are indicative of large-scale changes in the functional brain network for relapsing-remitting MS patients. The changes in the alpha2 band, such as loss of hierarchical structure, results in poorer cognitive performance. In the case of Parkinson’s, the observed studies reports common evidence for altered resting-state networks on a global, intermediate and local level in Parkinson’s patients. The Parkinson’s patients often show cognitive impairments, effective changes and other non-motor symptoms, suggesting system-wide effects on brain function. The functional graph analysis for both Parkinson’s and Alzheimer’s reveals that with the disease progression, the brain networks move towards a more random network organization.

#### 3.2.2. Analysis of Structural Brain Networks

The focus of most research that is based on the structural brain networks of neurological disorders is on the topological characterization and finding structural abnormality of the brain networks.

The most extensively-studied neurological disorder using structural networks is Alzheimer’s disease. A work by He *et al.* [83] reports the changes in the coordination of large-scale structural brain networks due to Alzheimer’s disease by considering cortical thickness data from structural MRI. The structural brain network that is constructed denotes the cortical regions as nodes and the physical connectivity of these regions as edges. The graph is analyzed using graph theoretical measures, such as the clustering coefficient, path length and betweenness centrality, to determine abnormalities in Alzheimer’s patients, which are associated with alterations in cortical thickness correlations, small-world parameters, nodal centrality and network robustness. It was observed that these variable values were larger in the Alzheimer’s brain graph as compared to the control cases. The results obtained were consistent with existing studies showing increased structural and functional asymmetry in Alzheimer’s patients that suggested that the widely-distributed cortical networks are altered in Alzheimer’s patients. Additionally, the analysis findings in the paper suggest that Alzheimer’s disease-related alterations in structural networks and their internal topology are biologically meaningful and are likely to explain the functional impairments associated with Alzheimer’s disease. Furthermore, Lo *et al.* [84] considered graph theoretical measures, including the clustering coefficient and shortest path length, and showed disrupted topological organization in the large-scale white matter structural networks constructed from diffusion MRI of Alzheimer’s patients. Information is processed in the gray matter (cortex and subcortical structures) and passed along the network via the white matter. The result shows an increased ratio of the characteristic shortest path length predominantly in the Alzheimer’s group. This may be due to the degeneration of fiber bundles for information transmission and suggests that the connections between cortical areas have been changed with less strength (reduced white matter integrity) or longer pathways (disconnection). Their work provided the structural evidence for abnormalities of systematic integrity in Alzheimer’s disease.

Structural brain networks have also been analyzed for schizophrenia. Bassett *et al.* [85] investigated the topological characteristics of the hierarchical structure (global, divisional and regional) of brain networks constructed from whole-brain anatomical networks constructed from structural MRI of 259 healthy individuals and compared those of 203 people with schizophrenia. Measuring the degree, path length, clustering and small-worldness of the brain network, they showed that people with schizophrenia had reduced hierarchy, loss of frontal hub and increased non-frontal hubs, as well as increased connection distance. The analysis shows for people with schizophrenia, highly clustered nodes are more evenly distributed in terms of their degree, and frontal hubs are less prominent. The finding indicates that the neuro-developmental abnormalities in schizophrenia specifically impact multi-modal cortical organization.

## 4. Need for Integrative Analysis on Large Graphs

Most of the studies that have been performed on neurological disorders use limited types of data to generate single layer networks. However, as neurological disorders are complex systems, integrative analysis that involves various data types over various dimensions is often more informative and will allow better analysis and predictions. However, the analysis of a graph for such a system may turn out to be incomprehensible due to the increased complexity representing heterogeneous information within the same view. Thus, the major bottleneck is in the advancement of the design of algorithms for constructing and analyzing graphs representing multiple levels of information from multiple data types to reveal and characterize the complex pathology of neurological disorders. One of the most information-preserving abstractions of the complex data is multi-layered graphs. However, the actual application of multi-layered graphs is not yet widely studied due to the infancy in the consensus representations and the development of analysis methodologies. In this section, we review existing methods for integrative methods on graphs and look at the possibilities of multi-layer graphs as frameworks for the integrative analysis of various data types.

### 4.1. Integrative Analysis for Single-Layered Graphs

One of the trends in data science, especially in bioinformatics, is the integrative analysis of various data types. However, existing studies that involve integrative analysis of neurological disease based on graph structures are limited. Following are two examples of the integrative analysis of bio-graphs and brain networks.

Multiple types of bio-networks can be analyzed together. In a work by Hwang *et al.* [86], the OMIM phenotype-gene relation of the disease phenotype similarity network, the human gene interaction network, the disease categorization and the molecular pathways were analyzed together to determine phenotype, gene clusters and their associations in Alzheimer’s patients. They found that association information can provide a global pathway activity view of human disease classes and can facilitate the understanding of the underlying molecular mechanisms of disease. The work reports finding TMED10, which is a newly predicted gene in the Alzheimer’s pathway. This gene leads to the production of amyloid beta peptides, which is a critical feature of Alzheimer’s disease. They analyze the newly-predicted member gene by providing a network view of the disease pathway for Alzheimer’s. They also considered two-way hierarchical clustering in order to predict disease phenotype cluster-gene cluster associations. They observed predicted associations between 20 disease phenotype clusters and 200 gene clusters (pathways). Some of the pathways are predicted to be associated with neurological and psychiatric disease classes, including the prion disease pathway and the MAPK pathway associated with neurological disease class.

Separate studies of structural brain networks and functional brain networks have and continue to advance our knowledge of neurological disorders. On the other hand, integrative studies can further advance our knowledge of the structural-functional interconnection of the brain components in neurological disorders. Rudie *et al.* [87] carried out structural and functional brain network analysis of autism spectrum disorders. They analyzed the structural and functional brain networks separately and integratively using Principle Component Analysis (PCA) of graphical features extracted from the networks. Their analysis starts by constructing structural and functional networks with the same set of nodes. The functional network was constructed using the whole-brain parcellation scheme discussed by Power *et al.* [88], which is based on a large meta-analysis of fMRI studies combined with whole brain functional connectivity mapping. After construction of both the functional and structural networks, six graph features were extracted, and structural and functional were examined separately and interactively. Following are the six graph theoretical properties: (1) clustering coefficient; (2) characteristic path length (3) normalized clustering coefficient (*λ*); (4) normalized characteristic path length (*γ*); (5) small-worldness represented as the ratio of *λ* to *γ*; and (6) modularity. The graph features of the functional network showed that the individual with autism spectrum disorder had a lower clustering coefficient for nodes within default systems and secondary visual areas. They displayed less robust modular organization implying less distinct communities and indicated higher nodal participation coefficients. A high level of global efficiency was also observed, which reflects a less organized or more random distribution of functional edges. The graph features of the structural network indicated a high level of local and global efficiency in both the typically-developed and autism spectrum disorder groups. An important finding shows that in the autism spectrum disorder group, modularity decreased at a slower rate. This finding is contrary to the findings reported by Hagmann *et al.* [89], where decreasing modularity and increasing global efficiency of structural networks with development are shown. In a separate analysis of ASD and controls, a similar level of correlation between raw measures of structural and functional connectivity was observed. However, after combining the structural and functional network properties and applying PCA, a reduced balance of local and global efficiency between structural and functional networks was reported in autism spectrum disorder, which displayed association with age and inversely related with autism spectrum disorder symptom severity. They observed weaker connectivity within visual (largely secondary areas) and sensorimotor systems, supporting more widespread alterations in functional connectivity. Their analysis shows that integrative analysis has the potential for unraveling the pathology of neurological disorders that may not be possible by individual analysis.

### 4.2. Integrative Analysis of Multi-Layer Graphs

In the previous subsection, we have shown that integrative analysis provided more incite into the neurological disorder compared to the analysis of a single type of data. We now show how multi-layer graphs can further aid in the integrative analysis process by first looking at the description of multi-layer graphs and the availability of analysis methods. Then, we review the applications of the multi-layer graphs in practice.

#### 4.2.1. Multi-Layer Graphs

Multi-layer graphs are used to represent the complex behavior of different types of entities or how the relationship of entities changes over different aspects, such as time. The graph constructed includes multiple sub-graphs and the layer of connectivity between them. The multi-layer graph represents a much fairer amount of information than individual layers separately. It gives a suitable framework for integrative analysis.

Kivelä *et al.* [90] wrote an extensive description of multi-layer graphs. In their description, the most general form of multi-layer graph allows each node to belong to any subset of layers and allow edges to connect any node in any layer. In addition, the multi-layer graphs can have a ‘multi-dimensional’ property that can include every type of data. That is, multi-layer graph MG, with L layers is given as MG_*L*_ = (G_1_, G_2_, …, G_*l*_, E_*inter*_, E_*intra*_ ), where L is the total number of layers, G_1_, G_2_, …, G_*L*_ are the subgraphs on each layer with n nodes. E_*inter*_ is used to denote inter-layer links, whereas, E_*intra*_ denotes the intra-layer links. Each sub-graph is defined as G_*l*_ = (V_*l*_, E_*l*_) where V_*l*_ = v_1_, v_2_, …, v_*l*_ is the set of nodes common in all of the subgraph, and E_*l*_ stands for the intra-layer connectivity for layer *l*. The adjacency matrix of each sub-graph is denoted by A_*l*_ ∈ (0, 1)^(*n*×*n*)^ [90]. In this general form of multi-layer graph, not only can different types of bio-networks be integrated, but also bio-networks with various brain networks can be integrated, as well. Although the definition of multi-layer graphs can be generalized to describe networks that combine bio-networks with brain networks and, maybe, even over time, the analysis of such complex system is not currently possible, especially for large graphs.

Tensors, high dimension arrays, are the most common form of representation for multi-layer graphs being studied. Although higher mode tensors are much more complex than matrix, *i.e.*, two-mode tensor, to analyze, they are still the simplest form of representation of limited types of multi-layer graphs. A three-modetensor, for example, can be describe as follows: the first two modes describe the node to node relationship, while the third mode can be an additional dimension, such as time or types of experiment. In this description, the set of nodes considered in each layer remains the same. De Domenico *et al.* [91] describes formulas for graph theoretical measures, including degree centrality, clustering coefficients, eigenvector centrality, modularity, von Neumann entropy and diffusion, for multi-layer graphs that can be represented in a three-mode tensor. In addition, tensor factorization methods [92,93], such as for clustering purposes, can also be used to analyze the multi-layer graphs.

#### 4.2.2. Existing Application of Multi-Layer Graph Analysis

The multi-layer graph finds its applicability in various domains, such as social networks, telecommunication networks, smart grids and biological networks. There are still several challenges involved in multi-layer graph analysis. There is still no consensus on the representation or recognized analysis approach. Although with technical challenges still remaining, the multi-layer graph is being recognized for being beneficial in organizing and analyzing disease simultaneously, which enables improvement of the decisions made by medical professionals and patients [94]. We examine a few applications of multi-layer graphs in biological data analysis and brain network analysis.

Multi-layer graphs are being recognized in systems biology applications. We look at just two among several. First, Salem *et al.* [95] considered a multi-layer graph to integrate the co-expression pattern of genes to find biological modules and showed improved performance in gene function prediction. Multiple independent gene expressions were integrated for module discovery and functional annotation. The multi-layer weighted graph was constructed by considering the topology and co-occurrence between the co-expression link. The biological modules were discovered using the edge-based graph clustering approach on the weighted link graph. A work by Didier *et al.* [96] showed the use of multi-layer graphs to identify communities from multiplex biological networks. Four biological networks were constructed from different sources of interactions between human genes or proteins. The biological networks were PPI, co-expression network, pathway and network of complexes. Here, each biological network corresponds to the different layers of the multi-layer graph. The community structure for individual layers was computed using a network modularity-based clustering algorithm [97]. Further, consensus-clustering approaches computed a unique community structure from the community structures obtained on each graph independently. They showed that the use of multiplex-modularity better recovers communities in heterogeneous density and missing data contexts.

The multi-layer graph is also used to model time-varying brain networks. Bassett *et al.* [69] modeled the dynamic reconfiguration of functional brain networks during learning using a multi-layer graph constructed from fMRI imaging brain data during cognitive processing. Using a multi-layer graph, they identified functional modules over short time intervals and characterized their changes over time. The study provides insight to expose the learning-induced autonomy of sensorimotor systems. It also uncovers a distributed network of frontal and anterior cingulate cortices whose disengagement predicted individual differences in learning. Moreover, the availability of neuroimaging modalities with different spatial resolution levels enables integrating the functional connectivity between brain regions that helps to figure out the disruptedfunctional connectivity based on the BOLD signal in neurodegenerative diseases, such as Alzheimer’s [70].

We can see that multi-layer graphs have been applied with improved performance for integrative analysis of various bio-networks and for analyzing time varying brain networks. This shows the potential success of multi-layer graphs for more complex forms of data combination, such bio-networks with brain networks, bio-networks of different data types over time or structural networks in combination with functional networks over time.

## 5. Discussion and Conclusions

In this review, we showed how complex biological networks are analyzed using graph clustering and other graph theoretical measures in the study of neurological disorders. Various neurological diseases, including Alzheimer’s, Parkinson’s and autism, are analyzed using biological, as well as brain networks. The analysis of these networks help to get a better understanding of the biological changes that contribute to the dysfunctional state in these diseases. For biological networks, we highlighted the different types of data used to construct the networks and showed the significance of the analysis of PPI, gene-gene association and biological pathways in the study of neurological disorders. In the case of brain image networks, we reviewed both structural and functional brain networks constructed from brain signals and images from experiments, including MRI, EEG, MEG and fMRI, and show their importance in understanding neurological disorders.

We showed that graphical analysis of individual data types in the form of single layer graphs is well studied; however, there is a need for the integrative analysis of several data types. We suggest that multi-layer graphs are a good data structure for integrative representations and analysis for obtaining better insight for complex neurological problems. Existing studies of multi-layer graphs in the analysis of neurological disorder are few. However, all are shown to improve the analysis. Thus, we can predict increased analysis performance in many more scenarios of multi-layer graphs. For example, multi-layer graphs can be used to model spatial temporal changes in the brain structural and functional networks for the resting state and during the execution of cognitive tasks. In a dynamic system, firing of an action potential in a neuron takes a few milliseconds; the plastic change in synaptic strength operates over time scales of minutes to hours; and the repair of cognitive function after brain damage occurs over the duration of years. The multi-layer graph data structure seems appropriate to conceptualize the brain graph dynamics of various time scales. In the generalized framework, each layer models the interactions of the system at time t, and the extracted time-varying graph metrics quantify the evolution of the topological properties across time. Moreover, additional modes can be added to incorporate structural and functional brain networks to determine the effects of structural topology on networks and dynamics. The computational studies have shown that brains’ structural and functional networks are intimately related and share common topological features [66], which show high promise in the success of the analysis. Similarly, bio-network analysis can benefit from the use of multi-layer graphs. Multi-layer graphs can integrate the existing bio-network with other sources of related molecular information, such as Gene Ontology, biological processes and pathways. These integrative association models may enable findings in neurological disorders that may not be possible in individual analysis. In the most complex form, multi-layer graphs can represent bio-brain networks, in which the biological network based on molecular data is combined with a network constructed from brain image/signaling data. The bio-networks are informative and reveal the genetic causality of the disease; on the other hand, brain networks help in determining the structural and functional changes of the human brain. Modeling of such a network could help with associating the genetic factors with the observed functional changes. The multi-layer graphs can help to capture the dynamic behavior of the network and allow integrative analysis of data. However, there are still several technical challenges, including efficient representation and inference algorithms, remaining in multi-layer graph analysis.

## Figures and Tables

**Table 1:** Graph theoretical measures for network analysis.

Measure	Scope	Computation
Clustering coefficient	Local	$c_{i}=\frac{2e_{i}}{k_{i}\left(k_{i}-1\right)}$, where $k_{i}$ is the degree and $e_{i}$ is the number of links between neighbors of the *i*-th node.
Local efficiency	Local	$E_{loc}=\frac{1}{N}\sum_{i\in G}E\left(G_{i}\right)$, where $E\left(G_{i}\right)=\frac{2}{N(N-1)}\sum_{i<j\in G_{i}}\frac{1}{d(i,j)}$, where $G_{i}$ is the subgraph of *G* that consists of node *i* immediate neighbors excluding *i* and d(i,j) is the shortest path length between nodes *i* and *j*.
Degree centrality	Local	number of edges emanating from a node
Betweenness centrality	Local	$b_{i}=\sum_{j,k\in N,j\neq k}\frac{n_{i,k}\left(i\right)}{n_{j,k}}$ where $n_{i,k}\left(i\right)$ is number of shortest paths between *j* and *k* that run through iand $n_{i,k}$ is the number of shortest paths between *j* and *k*
Closeness centrality	Local	$closeness\left(i\right)=\sum_{j}{\left[d\left(i,j\right)\right]}^{-1}$
Eccentricity	Local	$ecc\left(i\right)=\frac{1}{max\{d(i,j):j\in V\}}$ where *V* is the set of nodes
Radiality	Local	$rad\left(i\right)=\frac{\sum_{j\in V}\left({△}_{G}+1-d\left(i,j\right)\right)}{N-1}$ where ${△}_{G}$ is the value of the diameter
Characteristic path length	Global	$L=\frac{1}{N(N-1)}\sum_{i,j\in N,i\neq j}d\left(i,j\right)$
Global efficiency	Global	$E_{glob}=\frac{1}{N(N-1)}\sum_{i,j\in N,i\neq j}\frac{1}{d(i,j)}$
Minimum spanning tree	Global	Kruskal’s algorithms [20], *etc.*
Modularity	Global	$Q=\sum_{i=1}^{k}(e_{ii}-a_{i}^{2})$ where $e_{ii}$ is the fraction of edges that connects nodes in module *i*, $a_{i}^{2}$ is the fraction of edges that connect at least one node in the module *i* and *k* is the number of modules

**Table 2:** Biological network public resources. PPI, Protein-Protein Interactions.

Group	Name	Description	Uniform Resource Locator and Reference
PPI	Mint	Collects experimentally-verified PPIs in a binary or complex representation. Merged with InAct since 2013.	http://mint.bio.uniroma2.it/mint/ [27]
	String	The known and predicted protein interactions. The interactions include direct (physical) and indirect (functional) associations derived from genomic context, high-throughput experiments, coexpression, previous knowledge.	http://string-db.org/ [28,29]
	DIP	Manually- and automatically-curated database. Experimentally-determined interactions between proteins.	http://dip.doe-mbi.ucla.edu/dip/Main.cgi [30]
Biological Pathway	HPRD	Human PPI manually extracted from the literature.	http://www.hprd.org/ [31]
	KEGG	Manually-curated pathway maps representing knowledge of the molecular interaction and reaction networks.	http://www.genome.jp/kegg/ [32]
	Reactome	Manually-curated pathway.	http://www.reactome.org/ [33,34]
	Alz-Pathway	Manually-curated; comprehensively catalogs signaling pathways for Alzheimer’s disease.	http://alzpathway.org/ [35]
	Pathway-Common	Collection of publicly available pathway information from multiple organisms.	http://www.pathwaycommons.org/pc [36]
Gene Disease Network (GDAs)	DisGeNET	Integrated database from various expert-curated databases and text-mining-derived associations, including Mendelian, complex and environmental diseases.	http://www.disgenet.org/web/DisGeNET [37]
	CTDTM	Integrated chemical-gene, chemical-disease and gene-disease interactions manually-curated from the literature.	http://ctdbase.org/ [38]
Multiple Type	InAct	Standards-compliant repository of molecular interactions, including protein-protein, protein-small molecule and protein-nucleic acid interactions.	https://www.ebi.ac.uk/intact/ [39,40]
	BioGrid	Curated biological database of protein-protein interactions, genetic interactions, chemical interactions and post-translational modifications.	http://thebiogrid.org/ [41]
